# Molecular Detection and Genotyping of *Enterocytozoon bieneusi* in Environmental Sources near Cattle Farms in Korea

**DOI:** 10.3390/ijms26157270

**Published:** 2025-07-27

**Authors:** Haeseung Lee, Myungji Jo, Hyeyeon Kim, Kaifa Nazim, Seung-Hun Lee, Min-Goo Seo, Sang-Joon Park, Man Hee Rhee, Dongmi Kwak

**Affiliations:** 1 Veterinary Epidemiology Division, Animal and Plant Quarantine Agency, Gimcheon 39660, Republic of Korea; lhspppp@korea.kr; 2 Department of Veterinary Medicine, College of Veterinary Medicine, Kyungpook National University, Daegu 41566, Republic of Korea; jmj8161@knu.ac.kr (M.J.); sdcd33@knu.ac.kr (H.K.); koreasmg@knu.ac.kr (M.-G.S.); psj26@knu.ac.kr (S.-J.P.); rheemh@knu.ac.kr (M.H.R.); 3 Department of Veterinary Parasitology, Khalsa College of Veterinary & Animal Sciences, Punjab 143001, India; kaifa.nazim@gmail.com; 4 College of Veterinary Medicine, Chungbuk National University, Cheongju 28644, Republic of Korea; dvmshlee@chungbuk.ac.kr; 5 Institute for Veterinary Biomedical Science, College of Veterinary Medicine, Kyungpook National University, Daegu 41566, Republic of Korea

**Keywords:** *Enterocytozoon bieneusi*, water, soil, cattle farm, genotyping, zoonosis

## Abstract

*Enterocytozoon bieneusi*, a microsporidian protozoan parasite, infects diverse hosts, including humans and livestock. Transmission occurs primarily through the fecal–oral route or exposure to contaminated environmental sources, such as water and soil. While its prevalence in animals is well documented, data on environmental contamination—particularly in areas surrounding livestock farms—remain limited. Therefore, this study aims to investigate the presence of *E. bieneusi* in environmental sources near cattle farms in Korea, evaluating potential risks for zoonotic transmission. Overall, 364 environmental samples (soil and water) were collected from areas surrounding cattle farms and analyzed using nested PCR targeting the internal transcribed spacer region of *E. bieneusi*. One positive sample (0.3%) was identified in surface water near a shed housing Korean native cattle during autumn. Genotyping and phylogenetic analysis identified the sequence as originating from genotype BEB1, a Group 2 genotype commonly associated with ruminants and recognized for its zoonotic potential. While the detection rate was low, this represents the first report of *E. bieneusi* contamination in water near cattle housing and the first identification of BEB1 in environmental water in Korea. These findings highlight the potential for environmental transmission, emphasizing the need for further research and monitoring to inform strategies for public health and livestock biosecurity.

## 1. Introduction

*Enterocytozoon bieneusi*, an obligate intracellular microsporidian parasite, infects various hosts—including humans, livestock, and wildlife—and primarily causes gastrointestinal symptoms such as diarrhea, malabsorption, and weight loss, particularly in immunocompromised individuals [1,2]. The parasite is transmitted through environmentally resistant spores excreted in feces, which can persist in soil and water, facilitating fecal–oral and waterborne transmission [3]. Genotyping based on the internal transcribed spacer (ITS) region of the rRNA gene revealed the existence of numerous ITS genotypes clustering into distinct phylogenetic clades [3,4,5,6]. Group 1 comprises zoonotic genotypes found both in humans and in various animals, while Group 2 includes genotypes more adapted to ruminants. Some genotypes have been identified in both animals and humans, raising concerns about their zoonotic potential. Other groups (Groups 3–11, and possibly extending to Group 15 [7,8]) generally exhibit host specificity [4,6]. Zoonotic genotypes are widely distributed. For example, genotypes D and J (synonymous with BEB1), and Type IV—commonly associated with human infections— have been identified in sambar deer inhabiting water-catchment regions in wildlife studies in Australia [3]. Similarly, a study in Yunnan, China, found genotypes I, J, and BEB4 in cattle, all clustering within Group 2, while two other genotypes (YNDCEB-90 and YNDCEB-174) cluster within Group 1, indicating zoonotic potential [9].

Livestock serve as important reservoirs of *E. bieneusi* [10,11]. In Shanxi and Inner Mongolia, >34% of cattle tested positive for *E. bieneusi*, with genotypes such as J, I, BEB4, and BEB6—many within zoonotic Group 2—posing a significant risk to both animal and human health [12,13]. In Hainan Province, China, pigs and masked palm civets harbor multiple zoonotic genotypes, including D, EbpC, and EbpA, with one study reporting a 51.0% prevalence in civets [5,14]. These findings highlight the widespread distribution of zoonotic genotypes across diverse livestock-production systems. In addition, it has also been identified in various animals, including livestock studied in Korea [10,15,16,17,18,19,20].

Environmental studies further emphasize the significance of non-host reservoirs in the transmission of *E. bieneusi* [21,22,23]. Molecular detection in surface water and catchment areas reveals that environmental sources adjacent to livestock or wildlife habitats often contain zoonotic genotypes identical to those found in animals [3,24]. The persistence of spores in water and soil facilitates indirect transmission, thereby complicating control efforts. However, studies specifically targeting environmental matrices—such as soil and water—from livestock farms remain limited.

In Korea, the domestic livestock industry is growing rapidly due to the rising national income, changing dietary habits, increasing demand for livestock products, and supportive government policies. Beef is particularly favored, with a growing consumer preference for Korean native cattle. Cattle farms represent the largest segment of the domestic livestock industry in Korea, outnumbering the other types of livestock farms, including those for pigs, chickens, and ducks. These cattle farms are further classified into dairy and beef farms, with beef farms—primarily those raising Korean native cattle—comprising the vast majority and outnumbering dairy farms by a factor of 15 [25,26].

As the livestock industry grows, environmental concerns—particularly those related to management of livestock waste—have become increasingly significant, prompting the establishment of legal frameworks for environmental regulation. Despite advancements in farm infrastructure and environmental practices, waterborne zoonotic protozoa (*Cryptosporidium* spp., *Giardia* spp. and *E. bieneusi*) continue to cause outbreaks on livestock farms [27,28,29]. From a public health perspective, investigating the presence of these pathogens in the soil or in reservoir near livestock farms and evaluating the potential for contamination of surface water or groundwater is essential.

Therefore, this study aims to detect and genotype *E. bieneusi* in environmental samples (soil, surface water, and groundwater) collected near livestock farms. Additionally, the study seeks to identify zoonotic genotypes and evaluate potential pathways for environmental transmission by analyzing ITS sequences. The findings could help address critical gaps in our understanding of environmental reservoirs and contribute to a One Health approach for mitigating human exposure through environmental pathways related to livestock farming.

## 2. Results

### 2.1. Polymerase Chain Reaction-Based Prevalence

**Figure 1:**
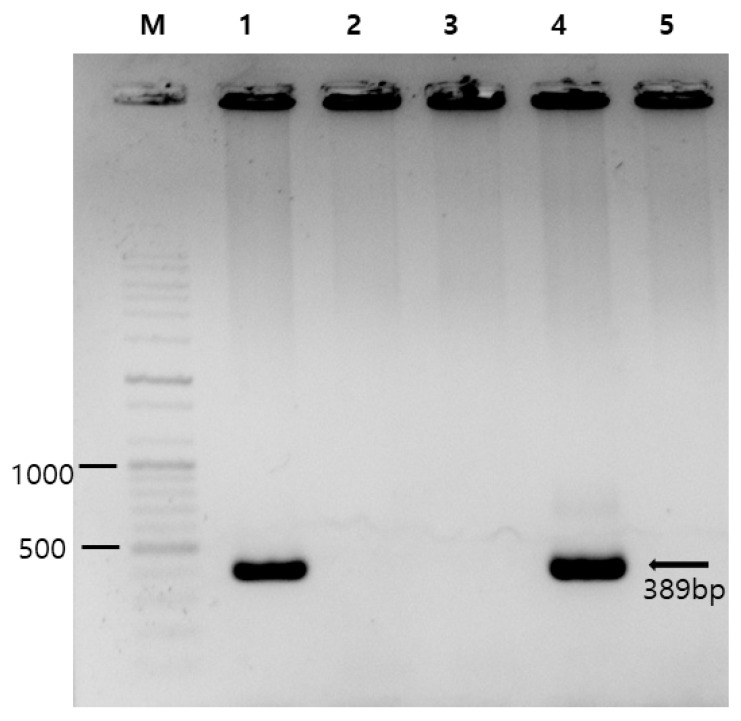
Photograph of gel electrophoresis used in PCR detection of *Enterocytozoon bieneusi*. Lane M, DNA marker (100 bp ladder); lane 1, a positive control (obtained from our previous study [10]); lanes 2 and 3, *E. bieneusi*-negative samples; lane 4, an *E. bieneusi*-positive sample (CCE58); and lane 5, a negative control (sterile distilled water). The numbers on the left are DNA size markers, and the arrow indicates the expected amplicon size of 389 bp.

Of the 364 samples collected, 124, 130, and 110 were from Chungcheong Province, Gyeongsang Province, and Jeolla Province, respectively. The overall detection rate was 0.3% (1/364) (Table 1, Figure 1).

A single positive sample was identified in Chungcheong Province (0.8%, 1/124). The seasonal distribution of the collection of the 364 samples was as follows: 76 collected in spring, 88 in summer, 120 in autumn, and 80 in winter. The single positive sample was collected during autumn (0.8%, 1/120). By farm type, 177 samples were collected near dairy farms and 187 samples near farms for Korean native cattle; the single positive sample was obtained from the latter (0.5%, 1/187). By sample type, 185 soil samples and 179 water samples were analyzed, with only one water sample testing positive (0.6%, 1/179). However, no statistically significant differences in positive test rates were observed across any of the variables.

### 2.2. Sequencing and Phylogenetic Analysis

**Figure 2:**
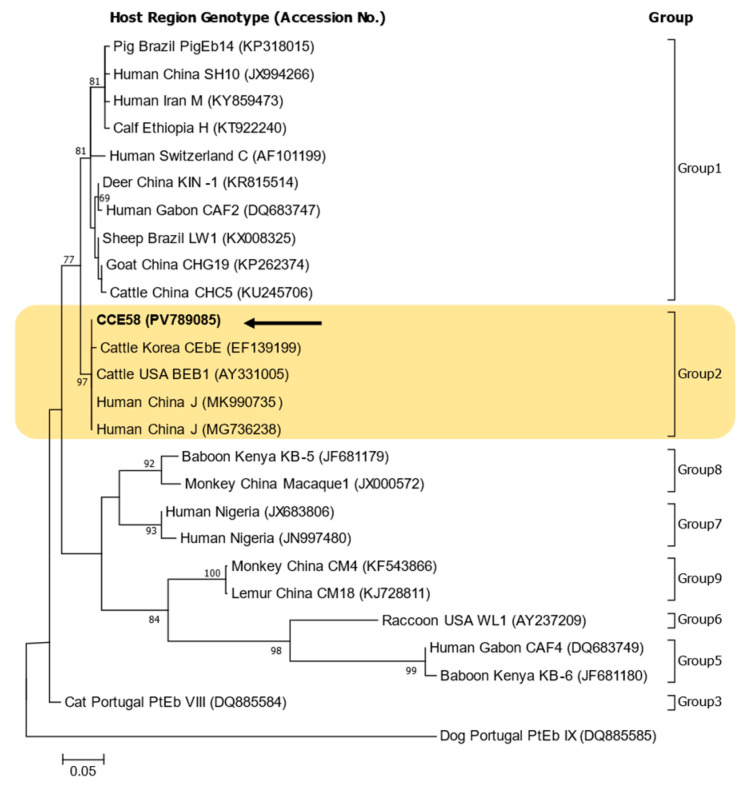
Phylogenetic tree of *Enterocytozoon bieneusi* genotypes based on ITS sequences. Sequences identified in this study are indicated by arrows. Reference sequences retrieved from GenBank are annotated with their accession numbers, host species, geographic origin, and genotype designations. All genotypes identified in this study clustered within Group 2. The numbers on the branches of the phylogenetic tree represent bootstrap support values as a percentage for 1000 bootstrap Replications. Scale bar represents 0.05 nucleotide substitutions per site.

Bidirectional sequences were obtained by sequencing analysis, and a consensus sequence was obtained using BioEdit version 7.7.1; this sequence was submitted to GenBank under the accession number PV789085. The 326 bp fragment contains small subunit ribosomal RNA and an ITS region. The sequence obtained from the polymerase chain reaction (PCR)-positive sample (CCE58) was classified within Group 2 of *E. bieneusi*. This genotype matched BEB1, which has previously been identified in cattle from both the USA and Korea (Figure 2).

## 3. Discussion

Our environmental surveys revealed a low prevalence of *E. bieneusi* (0.3%, 1/364). Although direct comparisons with the rates are limited due to the scarcity of studies focusing on environmental samples near livestock farms, the prevalence observed here is substantially lower than that reported in other studies. For instance, a Brazilian study reports higher positivity rates in raw sewage samples (16.6%, 3/18) and treated effluent (11.1%, 2/18) [30]. Similarly, a study from Mosul City, Iraq, reports the parasite in 8.6% (3/35) of surface water sources (canals and rivers) but not in any soil samples (0/20) [31]. Similarly, in our study, *E. bieneusi* was not detected from any soil samples. Soil samples often contain PCR inhibitors such as humic substances, which may reduce amplification efficiency [32]. We acknowledge that variable DNA yields and the presence of inhibitors may affect detection sensitivity, which remains a limitation of soil-based surveillance studies.

The prevalence of *E. bieneusi* in the Korean environment remains uninvestigated, although infection rates associated with other waterborne protozoa have been studied. In 1992, the rates of positive tests for *Cryptosporidium parvum* were confirmed to be 0.5% and 10.6% in Seoul and Jeonnam, respectively, with significant differences within Jeonnam between urban and rural areas, at 3.7% and 14.0%, respectively. The prevalence in Iyang-myeon, Hwasun-gun, Jeonnam, was particularly high (40.0%) compared to that in other regions, and a follow-up analysis confirmed a positivity rate of 35.2% (44/125). A high rate of *Cryptosporidium* positivity, 93.3% (14/15), was observed in cattle raised in the village, indicating that infected livestock may serve as a primary reservoir of contamination. Oocysts from cattle sheds are presumed to have entered the local groundwater system, triggering a community-wide outbreak among residents of Iyang-myeon, Hwasun-gun, Jeonnam who consumed the water. These findings suggest that environmental factors, such as the presence of soil and water near cattle sheds, may facilitate the infiltration of zoonotic protozoan into groundwater sources [33,34,35].

The potential for zoonotic pathogens to spread from animals to humans through environmental contamination remains a significant concern for public and veterinary health. Fecal matter from livestock, such as cattle, serves as a primary source of environmental pathogen contamination, facilitating the spread of *Cryptosporidium* oocysts, *Giardia* cysts, and *Enterocytozoon* spores into soils, surface water, and groundwater through runoff and leaching [36,37]. Therefore, *Cryptosporidium* spp. and *Giardia* spp. are included in routine water-quality testing, and this study provides baseline data on the presence of microsporidia. However, given the low microsporidia-positive rate of 0.3%, it currently poses minimal risk.

This pathway—from manure application in agricultural settings to soil and then to water bodies—illustrates a clear epidemiological chain exposing humans to waterborne zoonotic pathogens such as *Cryptosporidium*, *Giardia*, and *Enterocytozoon*. Breaking this chain requires a systematic One Health approach that integrates environmental surveillance, pathogen monitoring, and real-time data sharing across human, animal, and environmental health sectors [38].

Given the limited amount of data on the environmental transport of *E. bieneusi* spores, investigating how their small size facilitates contamination of surface water and groundwater is essential. Raising awareness of infection sources and transmission routes—particularly waterborne and foodborne pathways—guides effective prevention of urban *E. bieneusi* spread. Effective control and prevention of microsporidiosis requires a One Health approach that integrates medical, veterinary, and ecological disciplines through collaborative, interdisciplinary efforts [38].

The phylogenetic tree confirmed that the sequences obtained in this study belonged to Group 2. Previous studies in Korea have infrequently reported genotypes belonging to Group 2 [15,16]. There were five genotypes in bats (I, BEB8, KBAT1, KBAT2, and KBAT4) and four genotypes in cattle (CEbA, CEbB, CEbE, CEbF), and most of the others belonged to Group 1 (more than 20 genotypes) [10,11,15,16,17,18,19].

In previous studies focused on water-related sample, genotypes were identified as belonging to group 1 (SW1, SW2) or group 3 (WL6) or as an outlier (SW3) in stormwater samples from New York [23] and as belonging to group 1 (D, PigEBITS5, EbpA, Peru6, Peru8, Type IV, HNWW3 to HNWW5) or group 2 (BEB6, HNWW1, HNWW2) in wastewater samples from Zhengzhou, China [39]. In addition, studies in the water catchment area in Melbourne, Australia, identified Groups 1 (D, Type IV, MWC_d1, MWC_d2) and 2 (J) in wildlife [3] and Groups 1 (TAR_fc1) and 2 (BEB4, I, J, and TAR_fc2) in livestock [40].

For genotype comparison and construction of a phylogenetic tree, reference sequences included genotype BEB1 from USA cattle (AY331005) and genotype CEbE from Korean cattle (EF139199) [15]. The BEB1 (PV789085) sequence identified in this study showed 100% identity with the USA reference sequence (AY331005) and also matched sequences previously isolated from humans in China (MK990735, MG736238) [41].

The CEbB genotype (EF139196), previously isolated from Korean native cattle, matches that isolated from USA cattle (AY331005) and was excluded from the phylogenetic tree. However, compared to the co-isolated CEbE genotype (EF139199), it shows a single-nucleotide difference (317/318; A to G at position 98).

In addition to being used to construct the phylogenetic tree, the sequences identified in this study (PV789085) were additionally compared and analyzed with respect to the genotypes corresponding to Group 2 mentioned above. The genotype J sequence (MF693833) identified in deer in Australia also had a 100% sequence match (326/326) and was identical to a sequence from a sample detected in raw wastewater from China, which had also been previously isolated from cattle feces in Germany (AF135837).

Group 2 is primarily adapted to livestock, especially ruminants such as cattle, sheep, and goats. Genotypes BEB1, BEB4, BEB6, and I commonly occur in cattle and other ruminants and form the core of Group 2 [38,42]. Although they have been historically considered host-specific, Group 2 genotypes—particularly BEB1 and BEB6—have appeared in human clinical samples, suggesting zoonotic potential [7,43].

Consequently, Group 2 is primarily adapted to ruminants but is transmitted zoonotically and environmentally, especially where close human–animal contact or contaminated water exists. This understanding aligns with the One Health model and highlights its relevance in surveillance programs targeting livestock and public health.

Despite its low prevalence, detecting the zoonotic genotype (BEB1, Group 2) in the water near a shed housing Korean native cattle raises public health concerns. This marks the first report of BEB1 in environmental water in Korea, highlighting its significance. This study cannot confirm a direct link between farm-related contamination and the detected pathogen but highlights the need for further longitudinal and molecular epidemiological studies to trace contamination sources and assess public health risks.

The low number of positive samples limits the power of comparative analysis and thus explains the absence of statistically significant differences. Nevertheless, the findings underscore the importance of integrating environmental surveillance into existing frameworks for monitoring zoonotic disease. Future studies should include larger sample sizes, molecular typing of potential animal reservoirs, and quantitative microbial risk assessments to clarify the environmental pathways of *E. bieneusi* and its implications for livestock and human health.

## 4. Materials and Methods

### 4.1. Sampling Site and Period

Between July 2023 and June 2024, 364 soil and water samples were obtained from areas surrounding cattle farms in the central and southern regions of Korea.

The study required a minimum of 56 samples per region, as calculated using formulas from several literature sources [44,45,46], based on a 95% confidence level, 10% expected proportion, 10% absolute accepted error or precision, and the population size (number of farms). A statistically significant number of samples was collected. The population size (number of farms) was determined using the 2023 Q3 livestock data from the statistics compiled by Korean government [25,26]. According to national livestock-census data, Korea has approximately 88,000 Korean native cattle farms and an additional 5700 dairy cattle farms.

**Figure 3:**
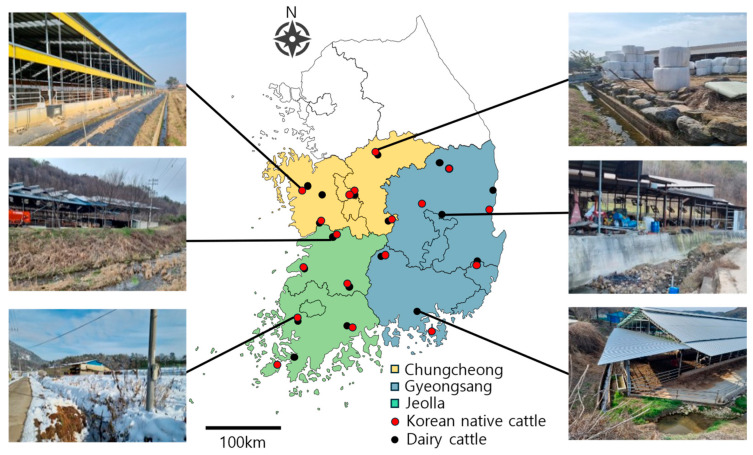
Geographic locations of sampling points. Sampling region (color shades) and site (red/black dot). Farm environment showing landscape (water and soil) surrounding some sampling points.

Administrative districts were grouped into three regions: Chungcheong, Gyeongsang, and Jeolla Provinces (Figure 3). From each province, six or more dairy and beef (Korean native cattle) farms were selected (Figure 3), and soil and water samples were collected from farms of each type. Sampling was conducted four times per farm—once in each season (spring, summer, fall, and winter)—to assess seasonal variation.

### 4.2. DNA Extraction and PCR

Water samples (10 mL each) were collected from each farm and concentrated via centrifugation (10,000 rpm, 10 min). The resulting pellets were used for DNA extraction. Soil samples (5 g) were washed and vortexed in distilled water for 5 min, and the pellets were used for DNA extraction using a QIAamp Fast DNA Stool Mini Kit (Qiagen, Hilden, Germany) according to the instructions of the manufacturer. DNA quantity and quality were measured with an Infinite 200 PRO NanoQuant plate reader (Tecan, Männedorf, Switzerland). Additionally, PCR amplification was conducted using AccuPower HotStart PCR Premix (Bioneer, Daejeon, Republic of Korea) and was followed by nested PCR targeting the *E. bieneusi* ITS region. The first-round PCR used ITSF1 (5′-GGTCATAGGGATGAAGAG-3′) and ITSR1 (5′-TTCGAGTTCTTTCGCGCTC-3′) as forward and reverse primers, respectively. The second-round PCR employed ITSF2 (5′-GCTCTGAATATCTATGGCT-3′) and ITSR2 (5′-ATCGCCGACGGATCCAAGTG-3′) as forward and reverse primers, respectively [20,47]. The expected amplicon size was 389 bp. PCR amplification was performed with a Mastercycler Pro (Eppendorf, Hamburg, Germany) under the following conditions: initial denaturation at 95 °C for 5 min; 35 cycles of denaturation at 95 °C for 30 s, annealing at 55 °C for 30 s, extension at 72 °C for 30 s, and a final extension at 75 °C for 5 min. All PCR products were visualized via 1% agarose gel electrophoresis with ethidium bromide staining (Figure 1), and positive samples were subsequently subjected to base-sequence analysis by Macrogen (Daejeon, Republic of Korea). To ensure the validity of the PCR and electrophoresis results, an *E. bieneusi*-positive DNA sample obtained from our previous study [10] was used as a positive control, and sterile distilled water was included as a negative control for visualization.

### 4.3. Phylogenetic Data Analysis

Sequence data obtained from the analysis were aligned using BioEdit and submitted to GenBank. For phylogenetic analysis, sequence data were retrieved from the NCBI Web BLAST search available online: https://blast.ncbi.nlm.nih.gov/Blast.cgi (accessed 1 June 2025). The phylogenetic tree was constructed using MEGA7 software version 7.0.26 [48], with bootstrap analysis performed with 1000 replicates.

### 4.4. Statistical Analysis

Statistical analysis was conducted using IBM SPSS Statistics for Windows, Version 26 (IBM Corp., Armonk, NY, USA). Chi-square tests were used to assess differences based on region, season, farm type, and sample source. A *p*-value of <0.05 was taken to indicate statistical significance.

## 5. Conclusions

*Enterocytozoon* is a waterborne zoonotic pathogen, and these characteristics highlight the need for baseline data to establish effective quarantine measures for livestock and public health.

In this study, the overall rate of detection of *Enterocytozoon* was 0.3% (1/364). Although there were no statistically significant patterns, positive cases occurred in samples collected from water near beef cattle sheds during autumn. Phylogenetic analysis revealed that these cases represented the BEB1 genotype, a Group 2 *Enterocytozoon* typically associated with ruminants but with recognized zoonotic potential. Despite the low prevalence, this study reports the first confirmed case of *Enterocytozoon* contamination in water near a shed and, particularly, the first detection of the BEB1 genotype in Korean environmental water.

A past incident in which oocysts from livestock facilities entered the groundwater system and caused community-level outbreaks serves as a critical lesson. The detection of *E. bieneusi* in environmental samples collected near livestock farms underscores the potential risk of exposure to waterborne zoonotic pathogens. These findings emphasize the necessity of adopting a One Health approach—integrating the human, animal, and environmental health sectors—to proactively prevent the urban spread of *E. bieneusi* and to respond more effectively to its potential public health consequences. While the contamination could not be definitively linked to the nearby shed, additional research and continuous monitoring are needed to track changes in contamination status.

## Figures and Tables

**Table 1:** Prevalence of *Enterocytozoon bieneusi* in environmental samples (soil and water) collected near cattle farms in Korea.

Variable		No. Tested	No. Positive (%)	*p*-Value
Region	Chungcheong	124	1 (0.8)	0.643
	Gyeongsang	130	0	
	Jeolla	110	0	
Season	Spring	76	0	1.000
	Summer	88	0	
	Autumn	120	1 (0.8)	
	Winter	80	0	
Farm type	Dairy cattle	177	0	1.000
	Korean native cattle	187	1 (0.5)	
Source	Soil	185	0	0.492
	Water	179	1 (0.6)	
Total		364	1 (0.3)	

## Data Availability

The original contributions presented in this study are included in the article. The newly generated sequences were submitted to the GenBank database and are openly available under accession number PV789085. The datasets used and/or analyzed during the present study are available from the corresponding author on reasonable request due to privacy considerations.

## Abbreviations

The following abbreviations are used in this manuscript:

ITS	Internal transcribed spacer
PCR	Polymerase chain reaction
Q3	Third Quarter
SPSS	Statistical Package for the Social Sciences
NCBI	National Center for Biotechnology Information
